# Anaesthetic Management of Conjoined Twins' Separation Surgery

**Published:** 2009-06

**Authors:** Kolli S Chalam

**Affiliations:** Head of Department, Anaesthesiology

**Keywords:** Conjoined twins, Separation surgery, Anaesthesia, Monitoring

## Abstract

**Summary:**

Anaesthesia for conjoined twins, either for separation surgery, or for MRI or other evaluation procedures is an enormous challenge to the paediatric anaesthesiologist. This is an extra challenging surgery because we the anaesthesiologists need to care for two patients at the same time instead of just one. Anaesthesia for conjoined twins ‘separation surgery mainly centered on the following concerns: 1.Conjoined Twins’ physiology like crossed circulation, distribution of blood volume and organ sharing with their anaesthetic implications. 2.Long marathon surgery with massive fluid shifts and loss of blood & blood components and their rapid replenishment. 3.Meticulous planning for organized management of long hours of anaesthetic administration in two paediatric subjects simultaneously with multi surgical specialties' involvement and their unique requirements. We report the anaesthetic and intensive care management of one pair of Pygopagus separation surgery and also the review of literature and world statistics.

## Introduction

Conjoined twins are identical twins whose bodies are joined in utero. It is a rare phenomenon; it is estimated to range from 1 in 50,000 births to 1 in 200,000 births^1^, with a somewhat higher incidence in Southwest Asia and Africa. They are identical twins (monozybotic and monochorionic) who develop with a single placenta from a single fertilized ovum. They are always the same sex and race. Approximately 75% of conjoined twin pairs are females. They are at a ratio of female to male 3:1. Of these, about 40% were stillborn, and 60% live born, although only about 25% of those that survived to birth lived long enough to be candidates for surgery. They are called miracle babies.

### Embryology

Two contradicting theories exist to explain the origins of conjoined twins. The older and most generally accepted theory is fission, in which the fertilized egg splits partially. The second theory is fusion, in which a fertilized egg completely separates, but stem cells (which search for similar cells) find like-stem cells on the other twin and fuse the twins together. However, rather than ‘fission’ or ‘fusion’, the defect leading to conjoined twins may well be a coalescence by overlapping of closely contiguous twin embryonic axis formative fields within a single embryonic disc (Potter, 1952 Willis, 1962; Beckwith, 2003^2^–^4^ It is likely that future understanding of embryonic induction and organizational centers may radically change how we envision the initial development of this complex anomaly.

It is presently thought that these factors are responsible for the failure of twins to separate after the 13th day alter fertilization. Conjoined twins can be artificially generated in amphibians by constricting the embryo so that two embryos form, one on each side of the constriction. There are no documented cases of conjoined triplets or quadruplets.

## Classification

Conjoined twins are usually classified by the point at which they are joined (the Greek word pagos, meaning “that which is fixed.”) There have been as many as three dozen separate types identified in the last century. The following basic classifications can be combined to more closely define individual cases.

**Craniopagus:** There is cranial union only; it has an incidence of about 2% of all conjoined twins. Various forms and orientations of fusion may be seen, with both neural and major vascular connections e.g., dural sinuses. Craniopagus parasiticus: A second bodiless head attached to the head. Dicephalus: Two heads, one body with two legs and two, three, or four arms (dibrachius, tribrachius or tetrabrachius, respectively.) Separation is possible; depending on how much of the brain is shared. There is high risk of brain damage.

**Thoracopagus including Xiphopagus:** Anterior union of the upper half of the trunk. This is the most common form of conjoined twins constituting approximately 35-40% of all conjoined twins. Babies face one another and have major junction at the level of chest, with conjoined hearts and livers as well as upper gastrointestinal (G.I) tract. Separation surgery depends on cardiac anatomy.

**Omphalopagus:** Joined at the chest or abdomen. Similar to thoracopagus twins, but in this case the twins do not share a heart. This is the second most common type of conjoined twins, representing 30-35% of the total. Highest rate of separation survival is reported in this class of twins. Usually, only the liver is involved. Because the liver can regenerate itself, this scenario is preferred. A combination of types 1, 2 and 3 is called Cephalothoracopagus or Janus.

**Pygopagus**. Joined at the sacrum, are about 19% of all conjoined twins. Separation is possible. The survival rate is hiah.

**Parapagus:** lateral union of the lower half, extending variable distances upward, about 5% of all conjoined twins. Heart sometimes involved. Life with artificial limbs is the result. Diprosopus: One head, with two faces side by side.

**Ischiopagus:** Anterior union of the lower half of the body, about 6% of all conjoined twins. Heart is not involved. They are joined at the pelvis. Separation is physically possible; however, excretion and sexual organs' impairment might result.

**Parasitic Twins:** Rare forms of conjoined twins, having different patterns.

1. Parasitic twins: Asymmetrical conjoined twins, one twin being small, less formed and dependent upon the other.
2. Fetus in fetus: Situation in which an imperfect fetus is contained completely within the body of its sibling.

**Conjoined Twins (misnomer-Siamese Twins)** are commonly conceptualized as entangled singletons, two individual people who are tragically ensnared. This conceptualization implies that their situation is unnatural and undesirable and demands rectification through surgical separation. There are very few hospitals with expertise and infrastructure to undertake conjoined twins ‘separation surgery in the world. Till now about 250 separation surgeries have taken place around the world. It is still viewed by public with awe and wonder.

## Case summary

**Fig 1:**
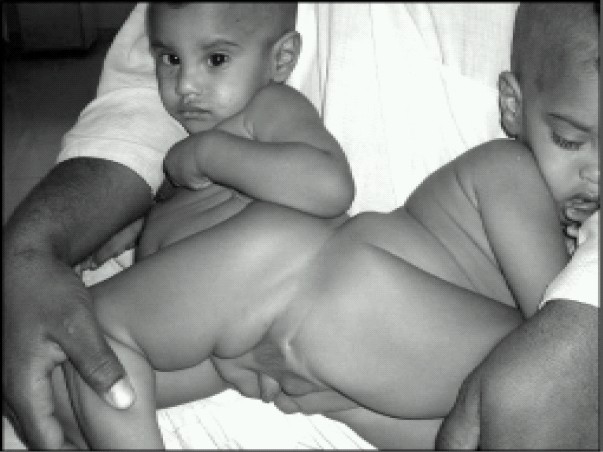
Pygopagus conjoint Twins

A pair of male Pygopagus conjoined twins aged 18 months from Sudan came to Abu Dhabi, the capital city of United Arab Emirates for the purpose of separation surgery in the month of June 2004. This pair is a healthy looking and charming boys. Their mother gave birth under a tree in rural Sudan(Fig 1).

**Fig 2:**
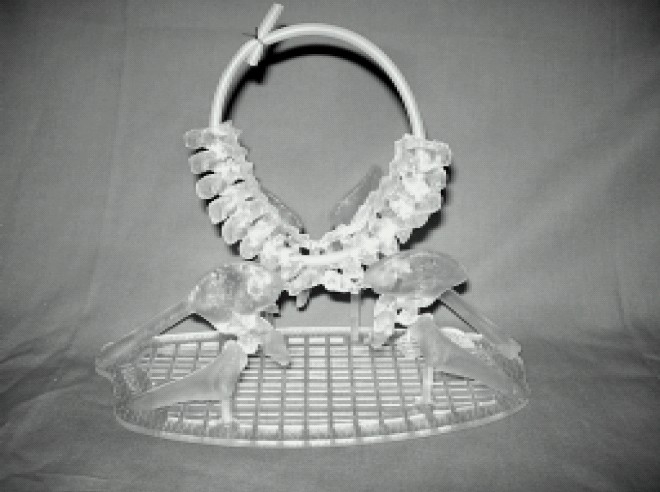
Computerized 3 D model of Pelvis.

Thorough preoperative evaluation was initiated that included routine blood and urine analysis, coagulation screen; plain x-rays, ultra sound scans, computed tomography scans, Digital substraction angiography (DSA), to delineate anatomic and bone detail, demonstrating organ position, shared viscera, and limited vascular anatomy. This particular set of twins has i) bony fusion at mid sacral posterior-lateral area. They have common spinal canal and thecal sacs, evidence of low conus and tethered cord with small syrinx within the inferior spinal cord of the twin B.

ii) Separate urinary bladders with a likely common urethra and common penis with 4 corpora cavernosa within the fused penis. Incidental bladder stone in twin B. iii) two separate rectums which appear to fuse distally near a common anal sphincter. iv) Bony fusion is along the mid sacral region of both the twins. At the level of the fusion numerous sacral ossification centers demonstrate atypical morphologies compatible with abnormal segmentation and separation. No other abnormality was noted. With the help of computer graphics 3 D model (computerized 3D virtual and physical workbench) was developed for surgeons to script the surgical sequence(Fig 2).

## Planning and rehearsals

Preoperative assessment and planning, with interdisciplinary communication and cooperation, is vital to the success of the separation operation. These twins require a dedicated team of anaesthetists for each child, and, consequently, duplication of all monitoring equipment in one operating room is necessary. Meticulous attention to detail, monitoring, and vigilance are mandatory. Planning for the postoperative period in the intensive care unit (ICU), as well as the babies' reconstruction and rehabilitation is essential from the time of the initial admission.

During the weeks required for expansion of sacralregion, representatives of the participating services about 40 personnel from Neurosurgery, plastic surgery, Urology, Pediatric Surgery, Pediatric anesthesiology, nursing, intensivists and operating room support staff met on several occasions to finalize the plan for the separation. The surgical plan, the anesthesia plan, staffing location of anesthesia and surgical equipment, patient positioning and plan for repositioning after physical separation were discussed.

An action plan for transfusion of blood and blood products was developed with the blood bank. The anaesthesia monitoring system in our largest operating theatre was modified to allow each anaesthesia team to orient and view both twins' vital parameters.

## Induction

Eight weeks prior to actual separation surgery plastic surgeons placed tissue expanders in the gluteal regions to gain skin expansion for closure of the raw area at the time of separation surgery. Two anaesthesia teams with two different anaesthesia work station and monitoring equipment administered anaesthesia with LMA placement three times for putting tissue expanders and their adjustments. The intra-operative course and recovery were similarly uneventful.

Prior to induction the presence of cross-circulation was tested with the administration of anti-cholinergic agent like atropine administered to one twin and no change in heart rate was observed in other twin. It appeared that there was no cross circulation between the twins. Sequential induction is advised when there is little or no cross circulation. But we carried inhalational induction supplemented with intravenous agents simultaneously as there is no significant cross circulation and both babies can be mask ventilated easily as they were facing opposite sides.

On the day of actual separation surgery i.e. 14.10.2004, the babies weighed together 20Kg and aged 21 months and heights were 68 cm and 72 cm respectively. They were wheeled into theatre after pre-medicating with glycopyrrolate 0.1mg, ranitidine 25mg and midazolam 2mg to each child. Dedicated teams of anaesthetists for each child and consequently, duplication of all monitoring and equipment in one operating room was organized to care them. Standard monitoring consisting of SpO2, EKG, and NIBP were instituted. Induction was carried simultaneously for both the babies with sevollurane, oxygen in nitrous oxide. Once they were sufficiently deep enough venous access with 18G cannula was secured on the dorsum of right hand in Twin A and left hand of Twin B and fentanyl 3 mcg.kg^−1^, propofol 2mg.kg^−1^, pancuronium 0.18mg.kg^−1^ were given. Mask ventilation and nasotracheal intubations were done sequentially. Nasotracheal intubation with 5.0 ID was chosen to optimize fixation of the endotracheal tubes as it was expected that the babies would be repositioned several times during, the course of surgery.

Radial artery and central venous lines utilizing left radial artery and right internal jugular veins with the guide of ultrasound were placed and connected to respective monitors. Arterial BP, CVP were monitored along with respiratory variables like RR, TV, Paw, and ABG. Urinary bladder was catheterized for urine output measurement and naso pharyngeal temperature probe for temperature monitoring, neuromuscular monitoring were also placed.

Antibiotics namely augmentin (clavulanated amoxicillin), gentamycin and metronidazole were administered following induction of anaesthesia. Aprotinin (Trasylol) 1ml.kg^−1^ i.e. 10,000 units per Kg body weight was also given to abate hyperfibrinolytic haemostatic disturbance leading to massive blood loss.

Maintenance of anaesthesia was carried out with oxygen 33% in nitrous oxide 66% and isoflurane 0.5-1.2% with additional top up doses of pancuronium and fentanyl infusion in the dose of 25 mcg/hour to each twin. Twins were ventilated with pressure control mode with a tidal volume of 90-110ml to each baby and respiratory rate of 14-18/min to achieve eucapnic levels. Peak and plateau pressures were maintained at 15-16 cm of water.

Fluids like D5 in 0.45% saline was used utilizing the formula of 4+2+1 ml per Kg body weight to replace the deficit. Ringer lactate was used as maintenance fluid. Third space losses were replenished with Ringer solution, Colloid (gelofusine) and blood (PRBC). Fluids and blood were pre warmed before transfusion. Even irrigation fluid was also pre-warmed.

Twins were draped and repositioned for the plastic surgical team to incise the skin and remove tissue expanders and excise excess old scar tissue and temporarily sutured the flaps of skin tissue. Plastic surgeons use the new skin tissue created by the tissue expanders to cover the raw area at the end of surgery.

Neurosurgeons then joined and severed sacral bony union and dural communication but CSF leaked before ligation. It was repaired under magnifying microscope. Pediatric and urology team assessed urogenital and colorectal anatomy and separated the four corpora of the united penis. Common rectum was divided. Posterior aspect of the urethra was also separated.

**Fig 3(a, b):**
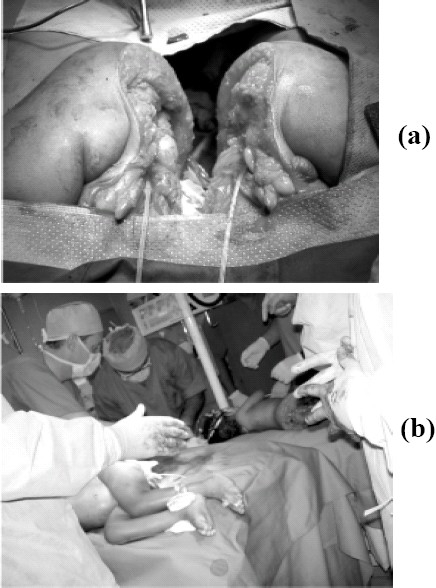
Intra-operative pictures

Pediatric surgical team actually separated twins at 18:30 hrs exactly after 6 and half hours after the onset of surgery. Two surgical teams consisting of paediatric and urology surgical teams carried out construction of separate Anii, construction of urethra, perineal body, pelvic floor, fixation of testicles into the corresponding hemi scrotal pouch and covering the shall of penis with appropriate skin on two separate operating tables with radiant heaters. The glutei were sutured to scrotal detect. Primary skin closure with suction drains by plastic surgical team with rotation flaps was accomplished. Completion of surgery took place by midnight nearly taking total duration of 20 hours(Fig 3a,b).

## Intra-operative course

Each twin needed three units of PRBC and 2.2 liters of colloid and crystalloid. Twin B required low dose dopamine of 5mcg.kg^−1^.h^−1^ and nitroglycerine infusion of 0.5mcg.kg^−1^.h^−1^ to maintain the filling pressures. It might be due to mild vasomotor instability. Mild hypokalaemia and hypocalcaemia needed correction. Heart rates were in the range of 108 to 128, mean BP in the range of 65 to 94 and CVP 6 to 12 cm of water and temperatures were 33.4 degrees Celsius at the end of surgery.

## Recovery and postoperative ICU care

Twins were shitted intubated, to ICU in separate trolleys for elective overnight ventilation. Morphine infusion was started in the dose of 20-30mcg.kg^−1^.h^−1^ as pain relief fin the ICU. Both were extubated the next day morning at 11.30am. Nitroglycerine and dopamine infusions were tapered and stopped. Both the babies maintained stable hemodynamics and good oxygenation. There was no neurological deficit observed. Hypospadias correction was advised to be carried later in Twin B.

Second postoperative day twin B developed fever to 39 degrees centigrade and seizures responded to tepid sponging, paracetamol, and 1mg midazolam, required respiratory assistance and so intubated and placed on ventilator. Dopamine and nitroglycerine were restarted to maintain pressures. Antibiotics were changed to cephatoxime, gentamycin and metronidazole. Fourth post operative day TPN was started with dextrose and amino acids and intralipid was added two days later. CT scan brain was carried on 5th post operative day showed mild ventricular dilatation and CSF leak was also found. Repair of CSF leak was carried out by neuro surgeons and skin was closed with rotation flap by plastic surgeons. CSF analysis and culture sensitivity showed enterococcus fecalis required vancomycin administration. This twin turned stable to be extubated on 6th postoperative day. Both the twins became fit to be shifted to general ward from ICU by 10th postoperative day.

**Fig 4:**
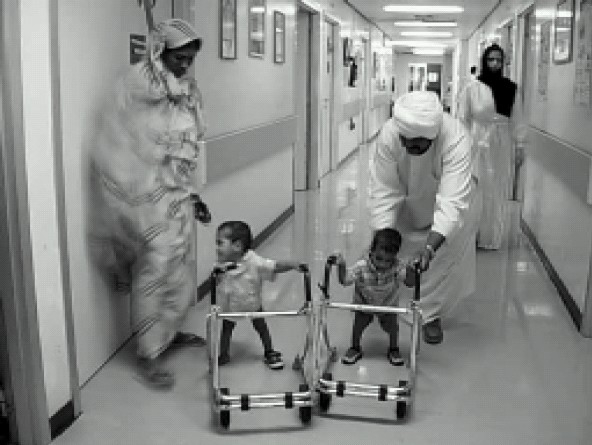
Separated twins moving freely

It was a scene for the news papers, TV channels and other public to take pictures and seeing them walking separately(Fig 4). This was the third pair who was operated in mafraq hospital, Abu Dhabi. Parents proudly carried their children each one separately in their arms and happily flew to their country of Sudan after spending a fortnight in general ward. They visited in subsequent years for repair of hypospadias correction and other minor procedures. But they are hale and hearty carrying on their routine.

## Comments

Conjoined twinning is one of the most fascinating human malformations. Treating conjoined twins can be a daunting challenge for the surgeon as well as anaesthesiologist. There are numerous conjoined twins in today's society. Surgical separations occur more frequently and with greater success than before. Surgery to separate conjoined twins may range from relatively simple to extremely complex, depending on the point of attachment and the internal parts that are shared. Most cases of separation are extremely risky and life-threatening. In many cases, the surgery results in the death of one or both of the twins, particularly if they are joined at the head, craniopagus.^5^ The decision of separation when dealing with conjoined twins is an extremely complicated one. It is a decision to be made solely by the parents. Separation is extremely risky. There is at best a fifty-fifty shot of survival when it comes to separation. If at all possible surgery seems like the best option for Conjoined Twins. Parents should make the final and informed decision on separation only after examining the physical, ethical, and psychological aspects of a possible separation.^6^

It is a multidisciplinary team approach involving extensive medical work-up on patients, multiple meetings and discussions with all the involved specialties and supporting staff, involvement of parents, Psychosocial counseling of parents, rehearsal of the planned surgical procedure, media contact prior to Surgery. The rationale for deferring surgery should include single heart, major communicating hearts or major anomalies.^7^

Goals of the anaesthesia care are to pay meticulous attention to detail, monitoring, and vigilance, planning for the postoperative care in the intensive care unit (ICU), by a dedicated team of anaesthesiologists and intensivists for each child with duplication of all monitoring and equipment in one operating room.^8^

Pharmacokinetics and pharmacodynamics are inconsistent in various types of twins. Usually there is more cross-circulation in the thoracopagus and craniopagus twins^9^ than in other types, and therefore one can expect altered and unpredictable drug responses.^10^ Estimation of circulatory mixing is useful to help calculate drug dosage and fluid replacement during surgery.^11^ Drugs administered to one twin may have unexpected effects on the other, especially for i.v. administration when circulatory admixing is present.^12^ Recommended i.v. doses of anaesthetic agents for the combined body weight of the twins are usually halved and then divided into two equal doses to be administered to each twin. Reduced incremental doses are titrated against response and help minimize the dangers of compounding drug effects in one twin.^13^

The routine evaluation of cross circulation is performed using many methods like Tc-99m microcolloidal human serum albumin (HSA), Tc-99m HIDA,^14^ injection of indigo carmine and the examination of its excretion in urine of the other twin.

However, if surgery for separation is planned, careful angiographic or radio isotopic imaging of the cross-circulation is necessary for estimation of the cardiac output percentage which is exchanged, as one of the twins might be dependent on the other's circulation for survival.^15^ It should also be recognized that the degree of cross-circulation is dynamic, highly dependent on both twins' relative systemic vascular resistance.

Szmuk P, Rabb MF, Curry B described the first use of bispectral index monitor for detection of cross-circulation in conjoint twins.^16^ In conjoint twins, synchronous ventilation is necessary to improve quality and decrease the time of the study.^16^ These authors decided to use the Carlens (Y) adaptor to achieve synchronous ventilation. Interestingly, the twins had different values of end-tidal carbon dioxide during the first hour and during the MRI scan (4-6 h), which equalized towards the end of anaesthesia. Authors believe that one reason for this disparity could be the differences in the compliance and resistance of lungs between the twins.

Though new technology advances in radiologic imaging, computer modeling, improved surgical and anaesthesia techniques enabled improved survival rates, age at operation influences success of Separation. It is suggested that surgery can be best delayed until such inlants are relatively mature (i.e., 6-12 months of age). Operative survival was 50% in those operated on in the neonatal period, but 90% in those over 4 months of age.

But 29-year-old female Iranian carniopagus twins (Ladan and Laleh Bijani) consented for separation surgery, believed to he the first fur adult craniopagus twins at Singapore's Raffles Hospital in 2003.^17^,^18^ The twins took 50 hours of anaesthesia. Dr.Carson of John Hopkins children's center joined the international efforts. It was unsuccessful. The twins died within 90 minutes of each other due to uncontrollable haemorrhage. The operation is more difficult in adults than in children whose brains are better able to recover from the surgery. Although Singapore doctors performed a similar operation in 2001 on infant Nepalese girls, surgery on adult twins is unprecedented.

In 1987, Benjamin Carson and a 70 member team successfully separated 7 month old Craniopagus boys from Germany. They were sharing superior saggital sinuses and so circulatory bypass, induced hypothermia and deliberate cardiac arrest was used to have better cerebral protection.^19^

Theodore G Wong et al of Singapore general hospital reported anaesthetic management and planning of the 5-day separation of 11-month-old craniopagus twins. The report emphasizes the importance of team-work, communication, and advanced planning required in twin separation surgeries that are rarely performed and have a long operating time.^20^

So, conjoined twins remain a topic of scientific speculation, public interest, and an image of two minds in the same body. Conjoined twins are increasingly accepted into our everyday lives as we grow to understand their unusual physical and emotional bonds and learn more about the science behind their development.
